# Pilot Evaluation of an Online Resource for Learning Paediatric Chest Radiograph Interpretation

**DOI:** 10.7759/cureus.12762

**Published:** 2021-01-18

**Authors:** Karthik Rajendran, Ben Walters, Bridget Kemball, Robert K McKinley, Nadir Khan, Colin A Melville

**Affiliations:** 1 Department of Trauma and Orthopaedics, Royal Free NHS Foundation Trust, London, GBR; 2 Department of Medicine, University Hospitals of North Midlands NHS Trust, Stoke-On-Trent, GBR; 3 Department of Medicine, University Hospitals Bristol and Weston NHS Foundation Trust, Bristol, GBR; 4 School of Medicine, Keele University, Newcastle-Under-Lyme, GBR; 5 Department of Radiology, University Hospitals of North Midlands NHS Trust, Stoke-On-Trent, GBR; 6 Department of Paediatrics, University Hospitals of North Midlands NHS Trust, Stoke-On-Trent, GBR

**Keywords:** online learning, radiology, feedback, deliberate practice, engagement, paediatrics, medical education, medical students, curriculum

## Abstract

Introduction and aims

Assessment of chest radiographs is a fundamental clinical skill, often taught opportunistically. Medical students are taught how to read adult chest radiographs, however, in our experience, there is often a lack of structured training for the interpretation of pediatric chest radiographs. Our aim was to develop and evaluate an online approach for medical students to learn this skill.

Materials and methods

Ericsson’s expertise acquisition theory was used to develop 10 sets of 10 practice radiographs which were graded using the X-ray difficulty score. Medical student volunteers (from Keele University School of Medicine) were recruited in the paediatric rotation of their first clinical year. Pre- and post-training tests of identical difficulty were offered. A semistructured focus group was conducted after the tests, the transcription of which was analyzed using grounded theory.

Results

Of 117 students in the year, 54 (46%) originally volunteered. The engagement was initially high but fell during the year, particularly during the pre-examination block. The high drop-out rate made the quantitative measurement of effectiveness difficult. The focus group suggested that pressure of other work, exam preparation, technical factors, and inflexibility of the study protocol reduced engagement.

Conclusions

Although the topic covered was seen as important and relevant to exams, the current system requires development to make it more effective and engaging

## Introduction

The interpretation of chest radiographs is an important skill for all medical students and doctors. It has been identified as a core skill both in the UK via the Royal College of Paediatrics and Child Health Undergraduate curriculum [1] and in the US via The Council on Medical Student Education in Pediatrics Clerkship Curriculum [2]. In our institution, it is taught opportunistically through individual cases, leading to variable acquisition of this important skill. Fewer opportunities are available for specifically learning to interpret paediatric chest radiographs. Therefore, we developed an online resource tailored for medical students to improve this skill

The resource is based on Ericsson’s model of skill acquisition, which emphasises the role of deliberate practice with immediate feedback [3]. Prior research has supported the hypothesis that radiograph interpretation improves using this method [4]. We hypothesised that systematic practice would improve students’ ability to interpret paediatric chest radiographs, with a dose-response effect. After the completion of the quantitative study, we conducted a student focus group to inform further development of the training resource.

## Materials and methods

Resource design

After reviewing relevant evidence [5-8], we considered the quantity of practice, the ratio of normal to abnormal radiographs, and the difficulty of the challenge to be key elements in designing the resource.

144 radiographs were chosen from those seen in clinical paediatrics (from a tertiary teaching hospital) over a 12-month period by an experienced Consultant General Paediatrician, and previously reported by a Consultant Paediatric Radiologist. They were selected to represent a broad range of pathologies, including respiratory, cardiac, gastrointestinal, emergency, orthopaedic, and child protection images. We included some normal radiographs (~25%) to ensure both normal variations and pathological radiographs were represented as previous studies [5-6] showed that between 50-70% abnormal films is an optimal mix of normal and abnormal radiographs for a training set [5-7]. Abnormal X-rays were randomly interleaved in each practice set, as this produces more improvement than grouped practice [8].

Each image was assessed using a published X-ray Difficulty Score (XRDS) ranging from -10 (most difficult) to +10 (easiest) based on a score of -2 to +2 for each of these five features: diagnostic difficulty, diagnostic confidence, typicality, subtlety, technical quality [6]. Scores were determined by five radiology trainees, five paediatric trainees, and two medical students, with mean scores calculated for each radiograph.

The final image set included 100 radiographs to cover the steepest part of the learning curve while also being manageable for students to complete [5].

Platform design

**Figure 1:**
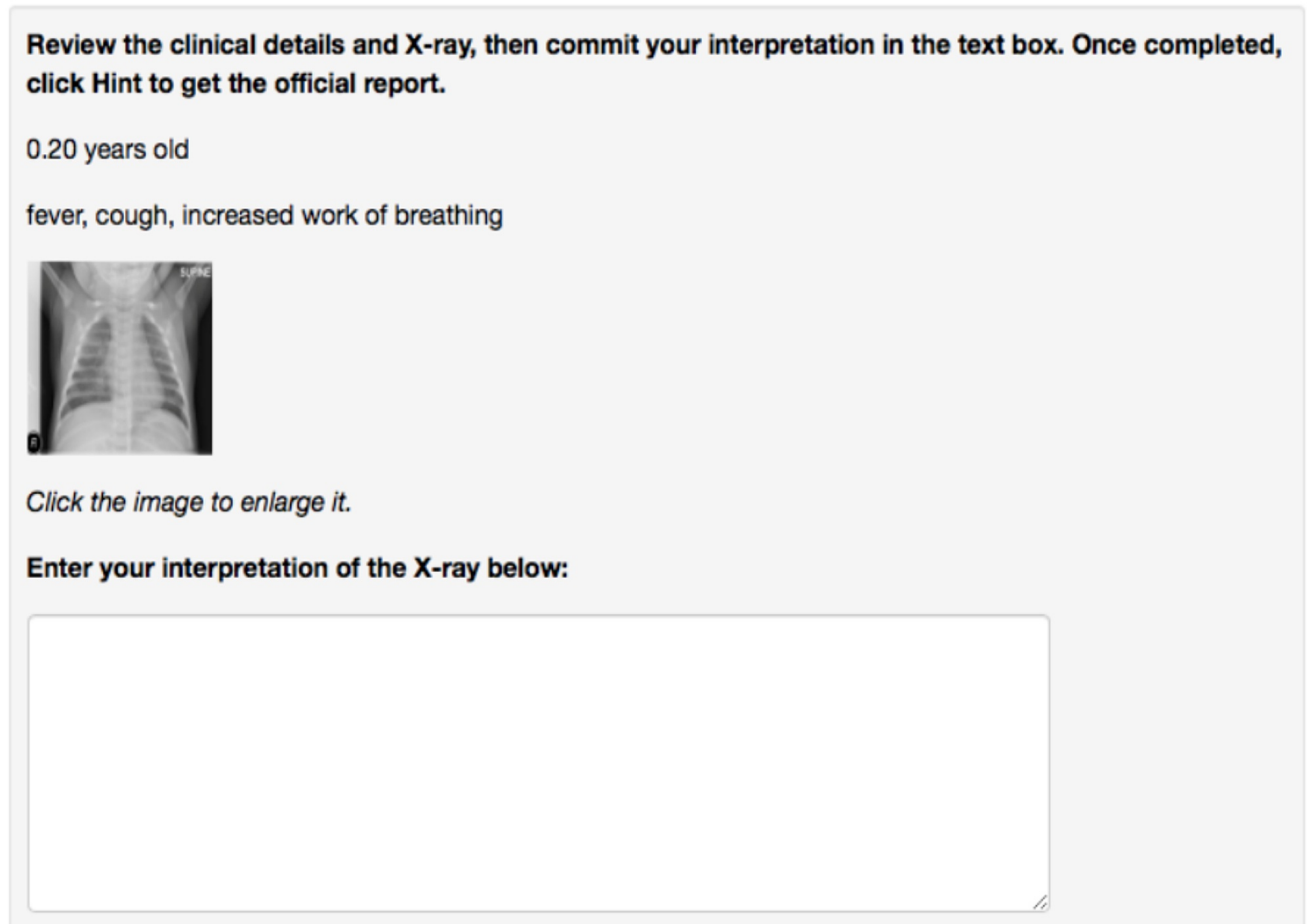
Online platform

The online user interface (Figure 1), or platform, presented students with radiographs in sets of 10. Each set contained normal and abnormal radiographs and was created to ensure that the films increased progressively in difficulty through the practice sets. This graduated challenge was intended to maintain motivation and interest with increasing skill [9]

In the 10 training sets, each individual film presents the age of the child and the original clinical details from the request form. The students reviewed each radiograph and wrote and saved their report, thus committing to their interpretation. They could then see the official report alongside their own interpretation, thus providing the student with immediate expert feedback. Each official report was approved by an experienced Consultant Paediatric Radiologist.

The platform also included three test sets of 10 radiographs. They were offered before, at mid-point, and at the end of training to measure the effectiveness of student learning. Each of the tests contained three normal and seven abnormal randomly ordered radiographs. The mean difficulty level of each radiograph was matched such that the overall difficulty score for each test was identical.

The platform was accessed through a web browser and hosted on a virtual learning environment. All students were assigned unique logins and passwords to access the site, which were generated and maintained by a member of the research team. All data from the system was anonymised by a single researcher who did not participate in the analysis.

Study design

*Learning Evaluation: *The study was approved by the University Ethics Committee. We used a two-group, non-randomised, single-blinded experimental controlled design. Third-year medical students on the Paediatrics and Elderly Medicine rotation at our institution were recruited to participate in this study. Half of the students had been assigned to Paediatrics first, and the other half to Elderly Medicine, thus providing the basis for the study groups. The evaluation consisted of three sets of test radiographs administered at four-week intervals during the Paediatrics/Elderly Medicine rotation. Students on the Paediatrics rotation first completed a pre-test with an immediate and delayed post-test. Those on the Elderly Medicine rotation first completed two pre-tests and a single immediate post-test.

Each student had timed activation and expiry to enable and disable access to the learning materials during the student’s Paediatrics block only and for each of the three tests. Each learning set reviewed the same radiographs in the same order. 

The primary evaluation measure was the proportion of students who engaged with the training material and the number of training sets of radiographs they reviewed (engagement, a Kirkpatrick Level 1 outcome [10]). We also planned to evaluate differences in student performance between test sets.

*Focus Group: *A focus group was convened after the completion of the evaluation study. The research team identified key topics to cover, which were used to guide the discussion. These included potential areas for improvement and issues related to participation.

Seven volunteers were recruited via an e-mail invite to the same third-year cohort by one member of the research team. Three of the volunteers had participated in the original evaluation of learning. The session was audio recorded and transcribed, then imported into NVivo 12 (QSR International Pty Ltd, Chadstone, Victoria, Australia) for analysis. The transcripts were coded by one member of the research team and grouped by emerging themes using grounded theory.

## Results

Learning evaluation

**Figure 2:**
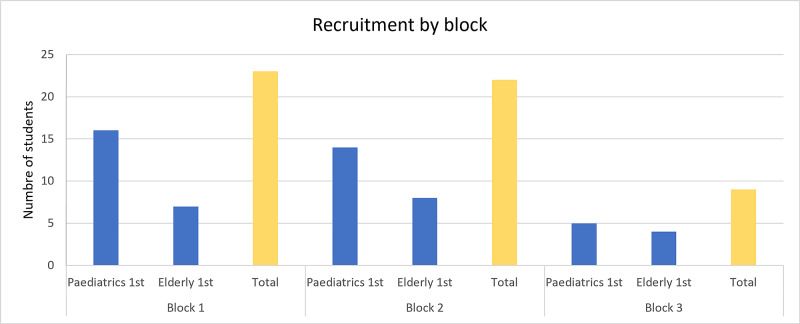
Recruitment by rotation

Of 117 third-year medical students, 54 (46.2%) consented to participate in the study (Figure 2). This consisted of 23 students in the first block, 22 students in the second block, and 9 students in the third block (prior to the end-of-year examinations).

**Figure 3:**
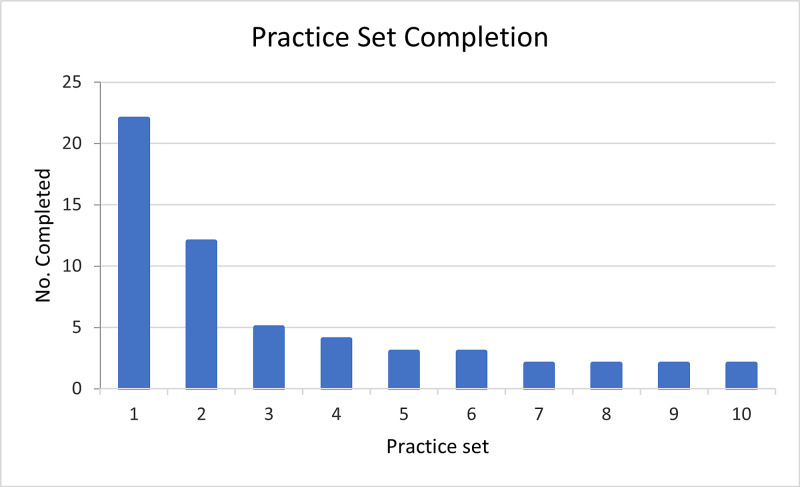
Completion rates for practice sessions

**Figure 4:**
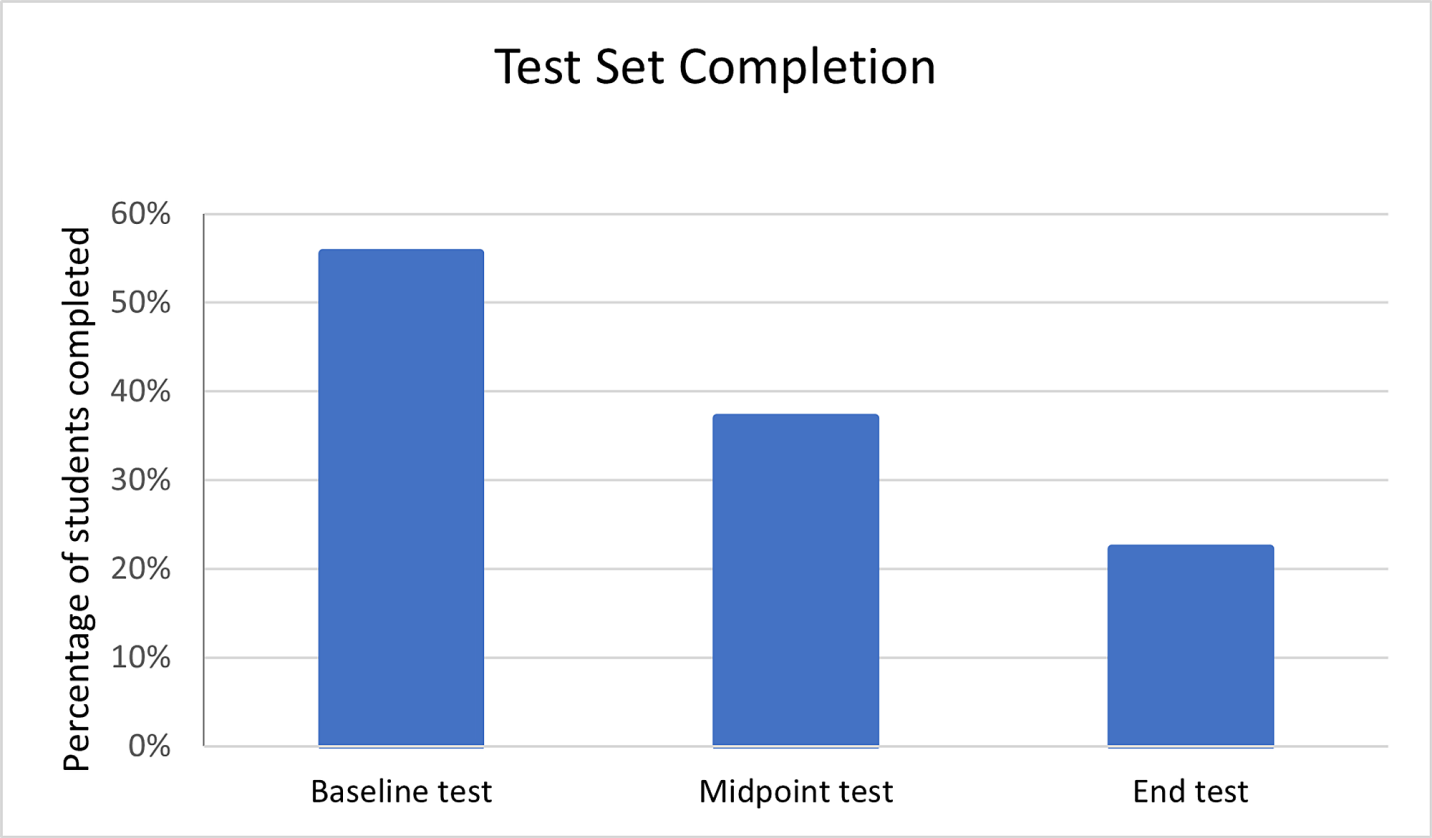
Completion rates for baseline, midpoint, and endpoint tests

Of the 54 students, 38 (70%) completed at least one test or practice set, with most completing the first practice set (Figure 3). There was a reduction in completion rates for the later practice sets (Figure 3). Some students completed no practice sets and only took part in the tests. With regard to the students who started doing a set, all but three students completed the ten questions. A similar trend was shown in completion of the tests, which showed the majority of participating students completing the initial tests (55%), followed by numbers decreasing for the subsequent midpoint (37%) and endpoint tests (22%) (Figure 4).

There was not enough data available to make meaningful statistical evaluations of students performance from baseline to midpoint to endpoint tests.

Focus group

Six major themes were identified, which are described below with illustrative quotes.

*1. Lack of Ward-Based Exposure: *“I was hoping I’d get from the wards and the teaching on the wards, I didn’t actually cover that enough to actually answer that question in the exam correctly” (ID6).

*2. Perceptions of Importance: *Although a part of the paediatric undergraduate curriculum, this is not often appreciated amongst students or doctors. “I was talking to my PDT (personal developmental tutor) about this actually. He said oh, you, you never see paediatric X-rays. Um, that she just thought it was a very strange thing to, for, for third year to be doing” (ID3). This is in contrast to the fact that paediatric radiographs form a part of undergraduate exams as highlighted by a few students. “And then it actually turned out that something similar to one of the X-rays that was on the programme actually turned up in the exam” (ID6).

*3. Transferable Skill:*Most students commented on the benefits they would gain in interpreting adult films in their adult medicine rotations. “I signed up because I thought it would be really good transferable skills cause reading a paeds X-ray is not gonna to be that different to reading an adult one” (ID2).

*4. Timing Within Medical School Curriculum: *There were mixed feelings from students about the implementation of the curriculum in the first clinical year. “I think if, if paediatrics slant in fourth year would have been, would have been good because then it, I think at that point you’ve probably got a bit more understanding of disease pathology, and I think things would be able to make a bit more sense to a student. Erm, especially after going through a whole year of erm, clinical medicine, and then interpreting it would be, or I feel it would be bit better” (ID4).

*5. Barriers to Participation and Completion: *Since there were both participants and non-participants in this focus group, reasons for non-entry into the study and reasons for not completing the curriculum were ascertained. One student found particular difficulty in joining on the programme: “I think I probably would have done it if, if there had been a, like, a direct, kind of, click on this, bang you’re in” (ID5). Workload of the primary medical degree was another factor: “So it was difficult to, to complete all of the exercise within that period alongside other, other studying that I had to do at the time” (ID4). The same student found issues with the platform itself: “I found that some of my responses weren’t being recorded properly” (ID4). The programme had strict deadlines, which some found difficult to adhere to: “I knew that there were deadlines, but I had actually missed the first deadline, erm. So therefore I was not able to complete the rest of the study” (ID7).

*6. Suggestions for Improvement:*** **A strong desire from the students was the provision of immediate feedback once a practice radiograph had been done. “And the consultant’s report was, it had gone into a lot of depth, and I found that useful because it made me, well it, it had already taken me to some other parts of the X-ray that I previously had ignored so” (ID4). Sending reminders out before deadlines was suggested, and this had an impact on students completion rates. “I’d forgotten about the deadline. I think they should have sent a reminder, or something” (ID2). One student mentioned e-learning about X-rays before doing the tests would have been beneficial when directly asked. “Yes, so maybe, like a little guide of what signs you might see” (ID2). Students seemed to be ambivalent about performance comparison amongst participants as a potential incentive to sign-up for the platform. “Actually performance measure against some of the cohort would get certain members of the cohort very into it [laughter], but that would only appeal, I think, to a certain mind set” (ID5). “It’s a double-edged sword because you, you could either look, look at the score and say oh, I’m top 10%, or oh” (ID2).

## Discussion

The initial recruitment of 46% of students highlights some concern regarding students' interest in leaning paediatric chest radiograph interpretation. Additionally, voluntary participation decreased in successive blocks of students. Only 2/54 (3.7%) students completed all 100 practice radiographs and all three tests, a dropout rate of >95%. In 2015, a study looking into the implementation and use of massive open online courses (MOOCs) across medical students in Egypt found a completion rate of 18.4%, with the majority of participants citing a lack of time as the primary reason [11]. Completion rates amongst MOOCs, in general, seem to be less than 10%, with differing reasons for the cause depending on the target population. Our completion rates, although low, are in line with these online experiences [12-13].

Potential reasons for this decline in participation include: the substantial study load of the course; competing academic priorities (e.g., student-selected component reports); exams and assignment deadlines; decreased enthusiasm for additional activities through exhaustion; that radiograph teaching is already covered through opportunistic learning; and that paediatric radiograph interpretation is non-core. These were explored in the focus group.

The focus group discussion supported the importance of this resource and suggested further developments. Students saw its relevance both in developing a clinically relevant transferable skill as well as being of relevance to exams, particularly given the limited and opportunistic radiograph teaching on wards. 

Students identified a number of factors relating to low participation rates. Single-click sign-up was suggested, which was not possible in the study for reasons of informed consent. One student did not appreciate the relevance at the time but regretted this when radiographs appeared in their exam. This suggests a gap between true and perceived syllabus content. The study design enforced hard deadlines, which sometimes were forgotten or clashed with other priorities. The perception of excessive numbers of practice films may have contributed to poor completion rates, as may commencement too near exam time.

Instant feedback was available, but, despite the written instructions, some students missed this, so a tutorial on using the system is under development. A further suggestion was an e-learning tutorial on chest radiograph interpretation. A recent study described using an e-learning module as an effective way to develop competency in interpreting radiological films [14]. With regard to the timing of the study, implementation in the first important element across all clinical medicine specialities, we feel this is the best period to incorporate the platform. This is supported by a study in preclinical medical students, which showed early radiograph exposure improved performance on tests of basic radiological knowledge compared with non-exposed students [15].

## Conclusions

This learning resource has the potential to help undergraduate medical students improve their paediatric chest radiograph interpretation skills. However, further work is needed to improve engagement, such as dedicated timetabled sessions during the paediatric rotation. This will also include integrated preparation and e-learning, a tutorial on the use of the resource, consideration of timing within the course, and flexibility of scheduling.
